# Prevalence of *Enterobius vermicularis* among children in Iran: A comprehensive systematic review and meta-analysis

**DOI:** 10.1016/j.parepi.2023.e00315

**Published:** 2023-07-13

**Authors:** Elnaz Moussavi, Mohammad Houssaini, Nader Salari, Mahvan Hemmati, Ahmad Abdullahi, Ali Asghar Khaleghi, Shamarina Shohaimi, Masoud Mohammadi

**Affiliations:** a Student research committee, Gerash University of Medical Sciences, Gerash, Iran; b Department of Biostatistics, School of Health, Kermanshah University of Medical Sciences, Kermanshah, Iran; c student research committee, Kermanshah University of Medical Sciences, Kermanshah, Iran; d Cellular and Molecular Research Center, Gerash University of Medical Sciences, Gerash, Iran; e Department of Biology, Faculty of Science, University Putra Malaysia, Serdang, Selangor, Malaysia

**Keywords:** *Enterobius vermicularis*, Enterobiasis, Pinworm, Children

## Abstract

**Background:**

Parasitic infections are among the most common diseases worldwide, and enterobiasis is a well-known type of parasitic infection in children. Given the existence of several reports on the prevalence of *Enterobius vermicularis* in different provinces of Iran and the heterogeneity of the reported prevalence data, this study aims to investigate the overall prevalence of *Enterobius vermicularis* among children in Iran through a systematic review and meta-analysis.

**Methods:**

This systematic review and meta-analysis study involved a comprehensive search of several databases, including PubMed, ScienceDirect, SID, and Google Scholar, focusing on cross-sectional studies that examined the prevalence of *Enterobius vermicularis* infection in Iranian children. The identified studies were entered into the EndNote software for review. The quality of observational studies was evaluated using the STROBE checklist. The information extracted from the studies was entered into the Comprehensive Meta-analysis (CMA, Version 2) software. Heterogeneity among the studies was analyzed using the I^2^ test, and publication bias was assessed using the Egger test and funnel plot.

**Results:**

A total of 51 studies, with a sample size of 46,070 children, were included in the review. Using the random effects method, the overall prevalence of *Enterobius vermicularis* among children in Iran was determined to be 6.7% (95%CI: 5.2–8.6). The review of the factors affecting study heterogeneity and sample size indicated that as sample size increased, the prevalence of *Enterobius vermicularis* among children in Iran also increased (*p* = 0.578). Additionally, with an increase in the year of conducting the studies, the prevalence of *Enterobius vermicularis* among children in Iran decreased (*p* < 0.05).

**Conclusion:**

The findings of this study show a relatively high prevalence of *Enterobius vermicularis* among children in Iran. We recommend health policymakers recognize the significance of this issue and take necessary measures to reduce the incidence of this infectious agent in children, implementing more effective preventive measures through mass media and educational campaigns.

## Graphical abstract

**Figure:**
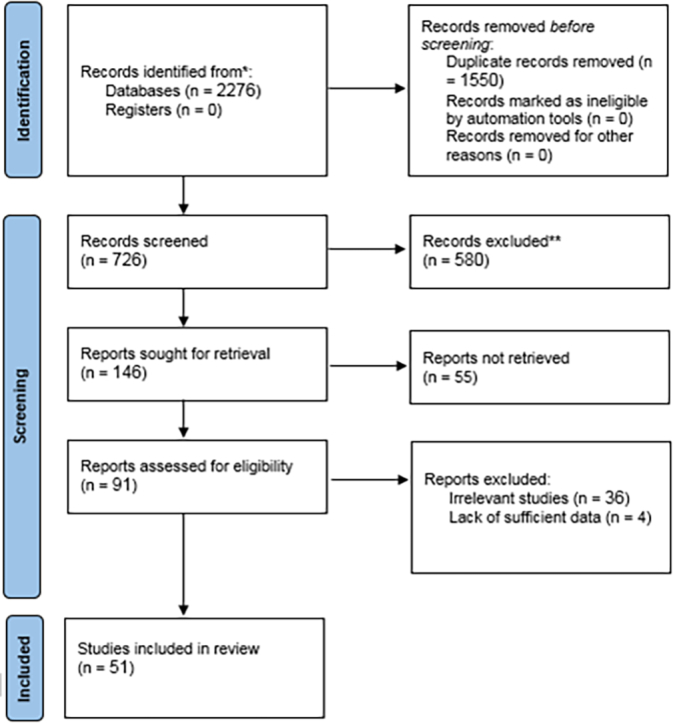

## 1 Background

*Enterobius vermicularis*, a species of parasitic worms belonging to the Oxyurida order, is responsible for causing enterobiasis (Fallah et al., 2022). These worms primarily inhabit the colon and migrate towards the anus, where they release approximately 10,000 eggs, facilitating the transmission of infectious eggs to other individuals, including self-reinfection, due to eggs' adhesive properties that cause irritation and itching in the anal region (Fallah et al., 2022; Fouladvand et al., 2018).

*Enterobius vermicularis* eggs can lead to infection through direct, indirect, and retro-infection transmission methods (Fallah et al., 2022; Haghi, 2013). In direct transmission, the eggs are transferred to food, drinks, or the mouth via contaminated hands, eventually reaching the ileocecal area and causing disease. However, their presence has been observed throughout the digestive tract, from the stomach to the anus (Fallah et al., 2022; Fouladvand et al., 2018; Haghi, 2013). The eggs are resistant to stomach acids and relatively resistant. To become infective, they require a temperature of 35 degrees Celsius for 4–7 h, while temperatures below 22 degrees Celsius induce dormancy (Fallah et al., 2022; Fouladvand et al., 2018; Haghi, 2013). *Enterobius vermicularis* infection manifests as anal itching, which can lead to sleep disturbances, restlessness, itching in the anal or vaginal area, irritability, teeth grinding, occasional stomach pain, and nausea (Fallah et al., 2022; Fouladvand et al., 2018; Haghi, 2013).

In indirect transmission, individuals at risk come into the infected person's clothing. Retroinfection, also known as double transmission, occurs when the remaining eggs around the anus re-infect the same individual (Haghi, 2013; Najafi et al., 2020). The severity of the disease in individuals is classified into two categories, severe and mild, based on the number of *Enterobiasis vermicularis* present in the body. In the mild type, fewer eggs are transmitted, usually through inhalation (Haghi, 2013). Due to their lightweight, the eggs can remain suspended in the air and infect individuals through inhalation or ingestion via the mouth or nose (Haghi, 2013; Najafi et al., 2020). Although practising proper hygiene, such as handwashing with soap, is generally recommended to prevent infectious diseases, it does not destroy *Enterobiasis vermicularis* eggs. However, exposure to sunlight and ultraviolet rays can destroy the eggs (Fallah et al., 2022; Fouladvand et al., 2018; Haghi, 2013; Najafi et al., 2020).

Parasitic infections, including enterobiasis, are prevalent worldwide (Fallah et al., 2022; Fouladvand et al., 2018; Haghi, 2013; Najafi et al., 2020; Rahim, 2013). It is estimated that approximately 400 million people globally are affected by enterobiasis, with earlier sources suggesting around one billion people being affected (Fallah et al., 2022; Fouladvand et al., 2018; Haghi, 2013; Najafi et al., 2020; Rahim, 2013). The prevalence of this infection is typically higher in temperate climates. Additionally, the level of personal hygiene in a society plays a significant role in the prevalence of enterobiasis. Developed societies with high literacy rates, awareness, industrialization, and the use of chemical fertilizers instead of human waste, generally experience lower prevalence rates compared to developing or less developed countries (Fallah et al., 2022; Fouladvand et al., 2018; Haghi, 2013; Najafi et al., 2020; Rahim, 2013).

In a developed country like the United States of America, which had a population of around 300 million in 2006, approximately 20–42 million people, or 7–14% of the population, were estimated to be affected by enterobiasis. In countries like Thailand or Sudan, much higher percentages, such as 38.5% and 37%, have been observed (Haghi, 2013; Najafi et al., 2020; Rahim, 2013).

Iran, a developing country with an infection rate of 17.2%, is at risk of a widespread epidemic of enterobiasis due to its geographical location and diverse climate. The prevalence of *Enterobius vermicularis* is higher in children compared to adults. Symptoms such as restlessness, loss of appetite, bedwetting, anal itching, teeth grinding, nightmares, stomach discomfort, nausea, and growth disorders cause significant suffering in children, leading to reduced productivity (Najafi et al., 2020; Rahim, 2013). The nocturnal migration of female worms for oviposition disrupts sleep, resulting in fatigue, difficulty concentrating and academic failure (Fallah et al., 2022; Fouladvand et al., 2018; Haghi, 2013; Najafi et al., 2020; Rahim, 2013).

Given that there have been several reports of the outbreak of *Enterobius vermicularis* in various provinces of Iran and the heterogeneous information available in published articles, this study aims to investigate the overall prevalence of *Enterobius vermicularis* among children in Iran through a systematic review and meta-analysis.

## 2 Methods

This systematic review and meta-analysis conducted a systematic search of selected databases. The articles retrieved were then screened, and studies meeting the predefined criteria were chosen by the authors. The relevant information from the selected studies was extracted, analyzed, and finally presented in accordance with the guidelines outlined in the PRISMA 2020 statement.

### 2.1 Search strategy

A systematic search was conducted across multiple databases, including PubMed, ScienceDirect, SID, and Google Scholar, to identify relevant articles for this study. The selection of keywords for the search was based on previously published primary studies and MESH Terms available in the PubMed database. The choice of keywords followed the PECO criteria, which included the studied population (children in Iran), exposure (*Enterobius vermicularis* worm), comparison (presence or absence of enterobiasis parasitic infection) and outcome (prevalence of enterobiasis parasitic infection caused by *Enterobius vermicularis*). The selected keywords were in English, while their Persian equivalents were used when searching in Persian databases. These keywords included comprised terms such as Enterobius, *Enterobius vermicularis*, Oxyuris vermicularis, Pinworms, Threadworms, and Children. Boolean search operators were used to combine these keywords effectively. The search was conducted without any time limitations, encompassing articles published until November 2022.

The search strategy of different databases is as follows:

((((((Enterobius[Title/Abstract]) OR (*Enterobius vermicularis*[Title/Abstract])) OR (Oxyuris vermicularis[Title/Abstract])) OR (enterobiasis[Title/Abstract])) OR (Pinworms[Title/Abstract])) OR Threadworms[Title/Abstract]) /Abstract])) AND (Child[Title/Abstract])) OR (Children[Title/Abstract])) AND (Iran[Title/Abstract]))))))))

### 2.2 Inclusion and exclusion criteria

This review focused on cross-sectional studies that specifically investigated the prevalence of *Enterobius vermicularis* among children in Iran. Studies such as case studies, case-control studies, cohort studies, clinical trials, systematic reviews, and meta-analyses were excluded from the analysis.

### 2.3 Study selection and data extraction

After collecting the identified studies using the Endnote software, the review process was initiated by the authors. The evaluations were conducted independently and in a blinded manner. Initially, two authors (EM and MH) assessed the title and abstract of each article based on the predefined inclusion criteria. In cases where there were discrepancies among the authors regarding the eligibility of an article, the final decision was made by a third party (MM).

### 2.4 Quality evaluation

The quality of observational studies was assessed using the STROBE checklist, which evaluates multiple aspects of study reporting. These aspects include the title, problem statement, study objectives, study type, statistical population, sampling method, data collection tool, and statistical analysis methods. The checklist comprises 32 items used to assess the quality of the studies. Based on these checklist items, each study was assigned a score ranging from 0 to 32. For this particular study, articles scoring 16 or above were considered as medium to good quality studies by the authors and included in the analysis.

### 2.5 Data analyses

The information extracted from the studies was entered into the Comprehensive Meta-analysis (CMA, Version 2) software. The heterogeneity among the studies was assessed using the I^2^ test, and the results were analyzed based on the heterogeneity determined by the random effects model. The presence of publication bias was investigated using the Egger test and Funnel plot. A meta-regression analysis was conducted to explore the factors contributing to the observed heterogeneity among the included studies.

## 3 Results

**Fig. 1:**
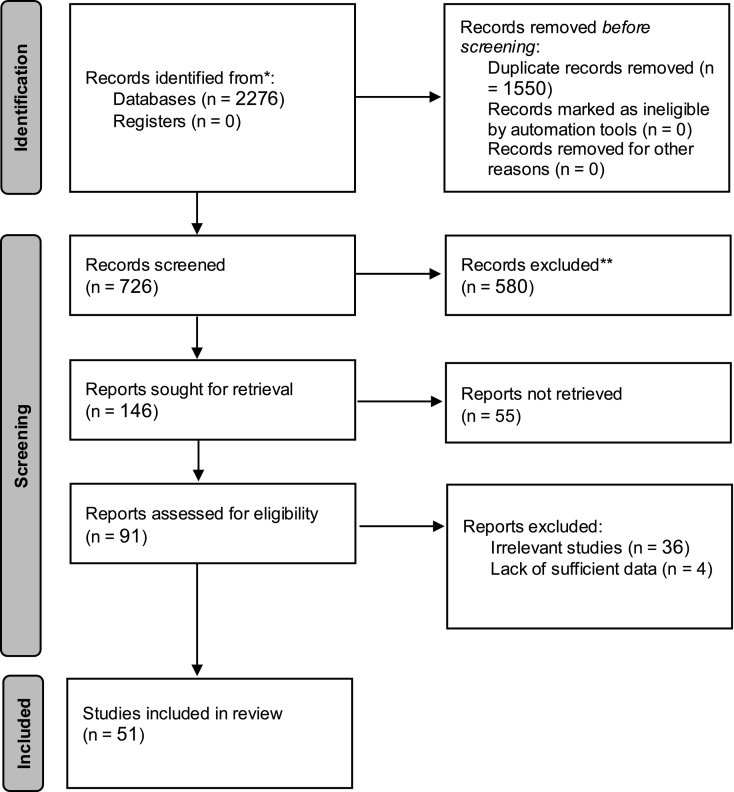
Identification of studies via databases and registers.

The search conducted across the analyzed databases yielded a total of 100 articles from PubMed, 134 articles from ScienceDirect, 1152 from Google Scholar, and 890 articles from SID. In total, 2276 articles were obtained from all the databases. After removing 1550 duplicate articles and further eliminating 580 articles based on the specified inclusion and exclusion criteria, 146 articles underwent a secondary evaluation. Finally, after excluding 36 articles unrelated to the subject and four articles lacking sufficient information, a total of 51 articles were included in the meta-analysis (Fig. 1 and Table 1).

**Table 1:** Extracted data from analyzed studies.

Row	Authors	year of publication	Location	age	Sample size	Prevalence
1	Fallah (Fallah et al., 2022)	2021	Hamadan	–	688	12.5
12	Foladvand (Fouladvand et al., 2018)	2018	Bushehr	–	203	3.1
27	Najafi (Najafi et al., 2020)	2020	Tehran	2–6	399	22.1
2	Zami (Zamini et al., 2016)	2002	Marivan	1–6	338	41.1
3	Fani(Fani et al., 2003)	2002	Gonabad	0–6	328	15.8
4	Moghimi (Moghimi and Sharifi, 2002)	2002	Yasouj	–	300	9
5	Taherkhani (Taherkhani and Sardarian, 2005)	2005	Hamadan	8.1	776	20
6	Yaghoubi (Yaghoubi, 2016)	2016	Gilan	–	599	4.01
7	Moshfea (Moshfea and Sharifi, 2000)	2000	Yasouj	–	612	23.2
8	Hazrati Tapeh (Hazratitape et al., 2007)	2006	Urmia	1–6	393	4.5
9	Hazrati Tapeh (Hazrati Tapeh et al., 2003)	2002	Urmia	1–6	830	35.4
10	Abbasi Fard (Abbasi Fard et al., 2004)	2004	Zahedan	–	415	4.7
11	Abbasi Fard (Abbasi Fard et al., 2004)	2004	Zahedan	–	438	15.5
13	Daryani (Daryani et al., 2004)	2003	Ardabil	–	400	3.1
14	Mouszadeh (Moosazadeh et al., 2017)	2017	Iran	–	11,676	17.2
15	Atashnafas (Atash Nafas et al., 2007)	2007	Semnan	–	688	17.2
16	Abedi (Abedi et al., 2004)	2004	Isfahan	–	451	0.4
17	Rahimi (Rahimi et al., 2015)	2015	Shahroud	–	261	2.3
18	Sharif (Sharif and ziaie hezar garibi H., 2000)	2000	Sari	2–5	811	2.1
19	Mousaviani (Mosaviani and Sc, 2006)	2006	Tehran	1–6	217	29.5
20	Amiri (Amiri et al., 2016)	2016	Babol	–	351	26.4
21	Sharifi Maud (Sharifi Moud et al., 2000)	2000	Zahedan	–	126	22.2
22	Afrakhteh (Afrakhteh et al., 2016)	2016	Amol	–	384	31.8
23	Kalantari (Kalantari and Mobadi, 2000)	2000	Babol	–	462	7.1
24	Azad (Soheili Azad et al., 2005)	2005	Tehran (Rabat karim)	–	368	33.6
25	Baghai (Baghaei et al., 2001)	2001	Isfahan (Mobarake)	under 12 years old	555	9.3
26	Taheri (Taheri et al., 2004)	2003	Birhand	6	650	16.1
28	Kosha (Koosha, 2006)	2006	Tehran	–	154	9
29	Nowrozi (Norouzi et al., 2016)	2016	Zanjan	–	1548	2.7
30	Shahabi (Shahabi, 2000)	2000	Tehran (Shahriar)	–	854	1.5
31	Rafiei (Rafiei et al., 2000)	2000	Tehran (Shahre ray)	–	1902	3
32	Mohammadzadeh (Mohammedzadeh et al., 1990)	1990	Tehran	–	1155	0.08
33	Mohammadzadeh (Mohammedzadeh et al., 1990)	1990	Tehran	–	254	0.4
34	Mohammadzadeh (Mohammedzadeh et al., 1990)	1990	Tehran	–	1060	0.85
35	Mohammadzadeh (Mohammedzadeh et al., 1990)	1990	Tehran	–	399	648
36	Ali Talari (Talari et al., 1998)	1997	Kashan	–	859	35.7
37	Ali Talari (Talari et al., 1998)	1997	Kashan	–	362	20.7
38	Eslami Rad (Eslami Rad et al., 1999)	1999	Arak	–	394	1.5
39	Hazrati tapeh (Hazrati Tappeh et al., 2010)	2015	Urmia	–	405	10.6
40	Ghahramanloo (Ghahramanlou et al., 2001)	2001	Babol	–	3429	0.7
41	Sharifi (Sharifi Sarasiabi et al., 2001)	2001	Bandar Abbas	–	1369	1.5
42	Badparva (Badparva et al., 2009)	2009	Lorestan	–	598	33.8
43	Farajzadeh (Farajzadeh and Foroughameri, 2003)	2003	Birjand	1–6	355	14.9
44	Ahmad Rajabi (Ahmad-Rajab et al., 2003)	2003	Bam	<7	370	15.9
45	Haji Alyani (Haji Aliani et al., 2014)	2014	Karaj	1–6	904	1.8
46	Khademi (Khademi and Arman, 2009)	2010	BandarAbbas	<8	534	0.9
47	Dawami (Davami et al., 2008)	2008	Jahrom	7–15	410	0.4
48	Mohammadvand (Mahmoudvand et al., 2020)	2005	Lorestan	2–15	366	6.8
49	Bahadori (Ranjbar-Bahadori et al., 2005)	2005	GhaemShahr	<9	2145	3.9
50	Torki (Turki et al., 2017)	2017	BandarAbbas	–	1465	0.1
51	Daryani (Daryani et al., 2012)	2021	Sari	7–14	1100	2.2

**Fig. 2:**
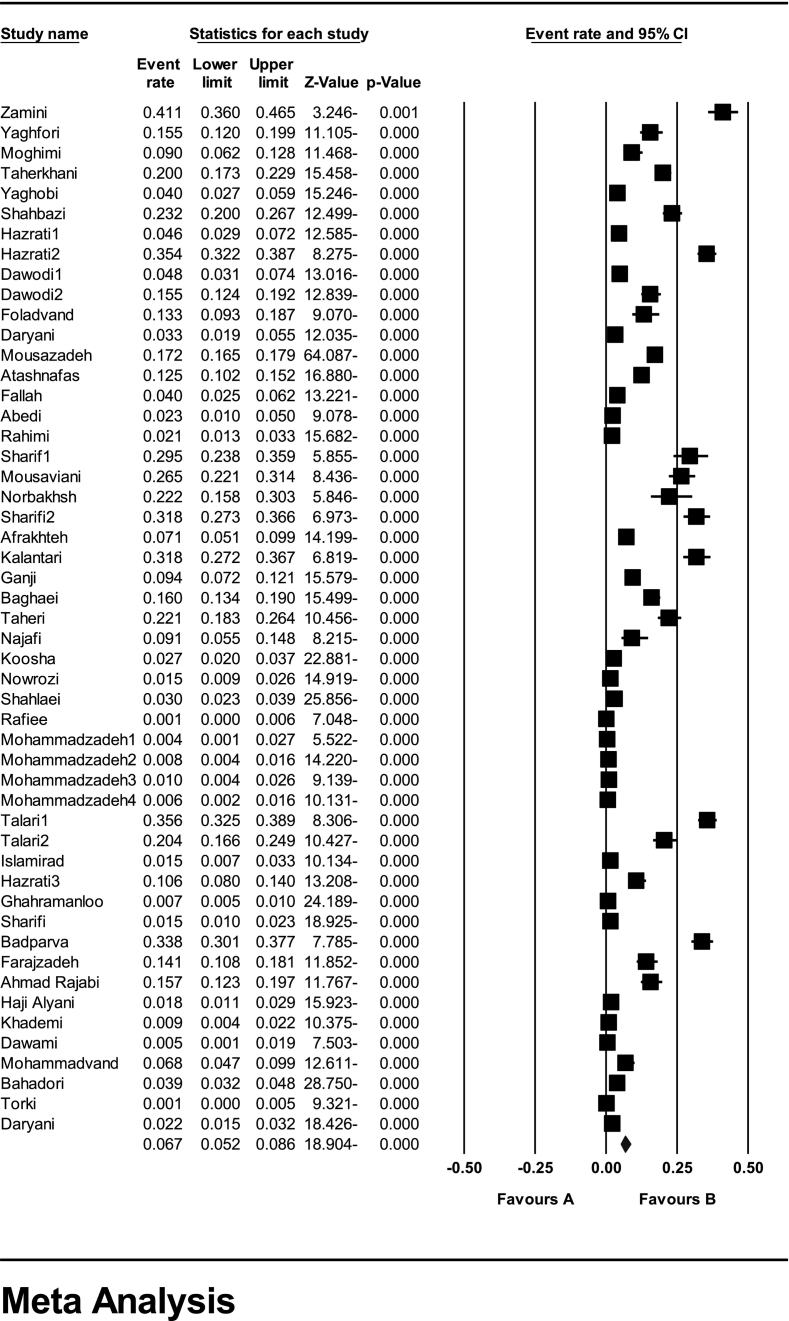
Forest plot of the prevalence of *Enterobius vermicularis* among children in Iran based on the random-effects method.

**Fig. 3:**
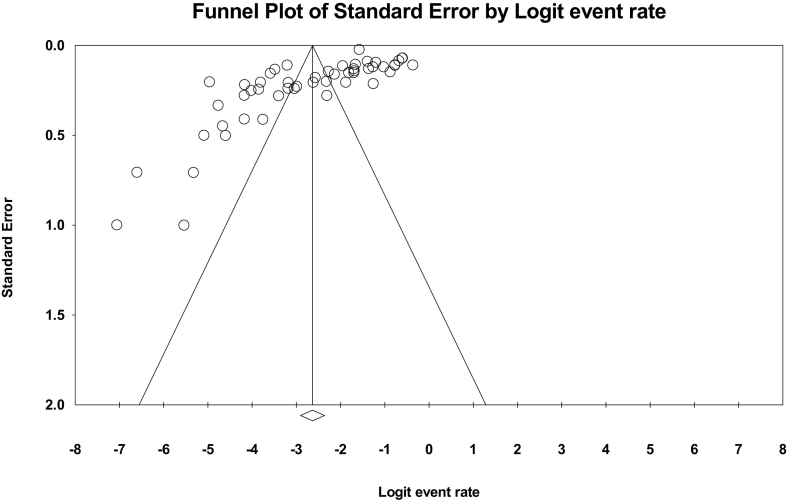
Funnel plot of the distribution bias in the reviewed studies.

In the systematic review of 51 studies involving a total of 46,070 children, the I^2^ heterogeneity test showed high heterogeneity (I^2^: 98.2). Based on this, the random effects method was used to analyze the results. The meta-analysis indicated a prevalence of *Enterobius vermicularis* in 6.7% (95% CI: 5.2–8.6) of children in Iran (Fig. 2). Furthermore, the presence of publication bias in the studies was assessed using the Egger test, indicating such bias's existence (*p*-value = 0.0003) (Fig. 3). As a result, caution should be exercised when interpreting the reported prevalence based on the meta-analysis.

**Fig. 4:**
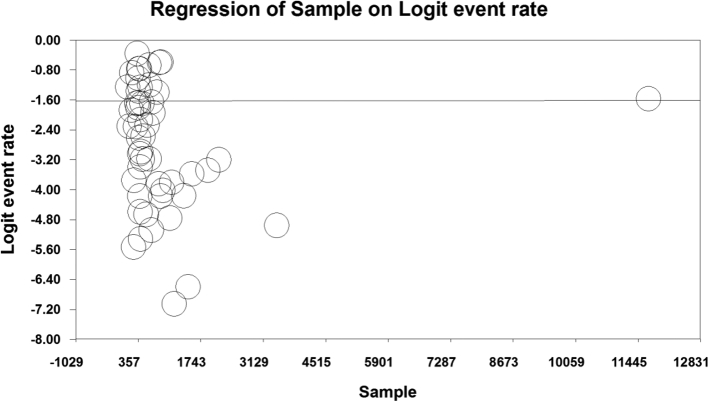
Meta-regression of the effect of sample size on the prevalence of *Enterobius vermicularis* in children.

**Fig. 5:**
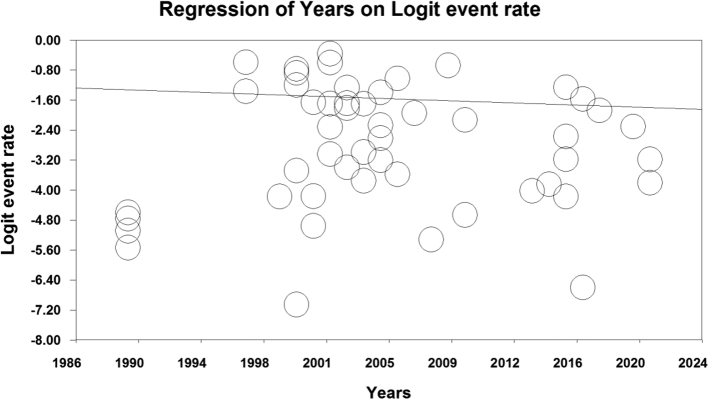
Meta-regression of the impact of the year of conducting studies on the majority of *Enterobius vermicularis* in children.

The investigation of factors influencing the heterogeneity of the studies and the effect of sample size on this heterogeneity revealed that as the sample size increased, the prevalence of *Enterobius vermicularis* among children in Iran also increased (p-value = 0.578) (Fig. 4). Additionally, an inverse relationship was observed between the prevalence of *Enterobius vermicularis* among children in Iran and the years of the conducted studies, indicating a decrease in prevalence over time (*p* < 0.05) (Fig. 5).

## 4 Discussion

According to the findings of this study, the prevalence of *Enterobius vermicularis* among children in Iran is reported as 6.7%. A previous study by Mouszadeh et al. in 2017 reported a higher prevalence of 17.2%. It is believed that the increase in the overall health level of society will lead to a decrease in the prevalence of this disease in the country (Moosazadeh et al., 2017).

Studies conducted in different regions have reported different prevalence rates for this parasite. For instance, a study in China by Li and colleagues in 2019 found an infection rate of 0.3% among the tested population of 45,427 individuals (Li et al., 2019). In India, a study by Latha Ragunathan and colleagues reported an infection rate of 1.9% among the participants (Ragunathan et al., 2010). Studies conducted in European cities such as Estonia and Slovakia reported 22.8% and 3.5% prevalence rates, respectively (Remm and Remm, 2008; Dudlová et al., 2018).

In Mexico, a study with a sample of 277 people found an infection rate of 18.3% among the participants (de la Luz et al., 2019). Another study conducted in Argentina reported a prevalence rate of 28.4% among the 303 participants (Rivero et al., 2018). In Ethiopia, a study by Tadege and colleagues found a % infection rate of 1% among the collected 600 samples (Tadege et al., 2022). In Egypt, a study by Elmonir et al. reported an infection rate of 8.6% among a total of 996 collected samples (Elmonir et al., 2021). Another report by Chia et al. found that 12.1% of individuals in the Marshall Islands had enterobiasis out of 346 samples collected (Fan et al., 2021).

*Enterobius vermicularis* is the only parasite from the Oxyurida family known to infect humans and cause various diseases. The presence of this worm in the human body directly leads to clinical symptoms of enterobiasis. This can result in fatigue, lack of concentration, reduced efficiency, and increased susceptibility to other diseases in children. *Enterobius vermicularis* is the only parasite from the Oxyurida family known to infect humans and cause various diseases. The presence of this worm in the human body directly leads to clinical symptoms of enterobiasis. This can result in fatigue, lack of concentration, reduced efficiency, and increased susceptibility to other diseases in children. Treating this infectious agent and the diseases it causes can impose significant costs on the healthcare sector (Rivero et al., 2018; Tadege et al., 2022; Elmonir et al., 2021; Fan et al., 2021).

Since this infection does not require an intermediate host, its transmission can occur rapidly. Neglecting personal and social hygiene contributes to its widespread occurrence and incurs substantial healthcare costs for treating the disease and its associated complications (Fouladvand et al., 2018). Factors such as the educational level and occupation of parents, place of residence, number of family members, and the social and economic status of the family, along with government policies, are believed to play influential roles in the spread of this infectious agent in society (de la Luz et al., 2019; Rivero et al., 2018; Tadege et al., 2022; Elmonir et al., 2021; Fan et al., 2021).

Implementing public education programs for children in kindergartens and schools, screening suspicious samples, prompt treatment and providing advice and health tips to parents can significantly reduce the likelihood of outbreaks in the community.

### 4.1 Limitation

The present study has several limitations, the most significant being publication bias in the reviewed studies. This bias affects the reliability of the results, and therefore caution is advised when interpreting them.

This study has comprehensively collected data from studies until 2022. By updating the results and using meta-regression analysis, the impact of two factors, namely sample size and year of publication, on the observed heterogeneity of 98.2% has been investigated. This approach enhances the reliability of the results and provides valuable insights for healthcare policymakers.

## 5 Conclusion

The present study shows a relatively high prevalence of *Enterobius vermicularis* among children in Iran. We urge health policymakers to recognize the significance of this issue and take necessary measures to reduce the occurrence of this infectious agent in children. Implementing effective educational programs and preventive measures through mass media channels is important to address this public health concern more efficiently.

## Ethics approval and consent to participate

Ethics approval was received from the ethics committee of the Deputy Vice Chancellor of Research and Technology Gerash University of Medical Sciences (IR.GERUMS.REC.1401.012). All methods were performed in accordance with the ethical standards as laid down in the Declaration of Helsinki. The present study was a review, and informed consent was not required.

## Consent for publication

Not applicable.

## Funding

By Deputy for Research and Technology, Gerash University of Medical Sciences (IR) (401000018). This deputy has no role in the study process.

## Authors contributions

MM and NS contributed to the design and MM statistical analysis and participated in most of the study steps. MM, EM, MmH, AA, AK, MvH, and SHSH prepared the manuscript. All authors have read and approved the content of the manuscript.

## Declaration of Competing Interest

The authors declare that they have no conflict of interest.

## Data Availability

Datasets are available through the corresponding author upon reasonable request.
